# Subjective personal and collective benefits of alcohol use in UK trans and gender diverse communities: a cross-sectional survey

**DOI:** 10.1093/alcalc/agaf075

**Published:** 2025-12-01

**Authors:** Dean J Connolly, Joseph Messinezis, Gail Gilchrist, Beth Thayne, Stewart O’Callaghan, Emma Davies

**Affiliations:** Addictions Department, Institute of Psychiatry, Psychology and Neuroscience, King’s College London, 4 Windsor Walk, London, SE5 8BB, United Kingdom; Department of Health Services Research and Policy, Faculty of Public Health and Policy, London School of Hygiene and Tropical Medicine, Keppel Street, London, WC1E 7HT, United Kingdom; Addictions Department, Institute of Psychiatry, Psychology and Neuroscience, King’s College London, 4 Windsor Walk, London, SE5 8BB, United Kingdom; Addictions Department, Institute of Psychiatry, Psychology and Neuroscience, King’s College London, 4 Windsor Walk, London, SE5 8BB, United Kingdom; Quouch, Luisenstraße 53 10117 Berlin, Germany; OUTpatients, 92-94 Wallis Road, London, E9 5LN, United Kingdom; Centre for Psychological Research, Oxford Brookes University, Headington Campus, Oxford, OX3 0PB, United Kingdom

**Keywords:** alcohol, gender diverse, non-binary, pleasure, transgender, United Kingdom

## Abstract

**Aims:**

This study sought to understand the subjective individual and collective benefits of alcohol use for transgender and gender diverse (TGD) people in the UK.

**Methods:**

A cross-sectional survey, co-produced with a TGD community advisory group, was open for responses from 1 February until 31 March 2022. Respondents were UK-based TGD adults who reported current or historical alcohol use. Those included in this sub-sample (*n* = 295) provided a valid response to one of two open-ended questions (OQ) concerning the subjective benefits of alcohol to TGD individuals (OQ1) and the perceived role of alcohol in UK TGD communities (OQ2). Data from these questions were collated (*n* = 455 responses) and coded using thematic analysis.

**Results:**

An overarching theme was identified, ‘belonging’, under which two themes were developed to answer each question. For individuals, alcohol enhanced intimacy and relieved pain, and was described to have a role in bringing the TGD community together and facilitating gender exploration and affirmation.

**Conclusion:**

TGD people experience a range of benefits from using alcohol, attributable to its anxiolytic and analgesic properties. Many respondents felt alcohol had a specific role in their communities, associated with the relative safety of alcohol-serving venues for TGD people.

## Teaser

**Short Summary:** UK-based trans and gender diverse survey respondents reported that alcohol fostered a sense of belonging by facilitating intimacy and relieving distress or pain that enabled social participation. Some, though not all, respondents reported that alcohol-serving venues played a role in bringing their community together and creating circumstances for gender affirmation.

## Introduction

A substantial body of research suggests that transgender (trans), non-binary and gender diverse (TGD) populations may experience a greater burden of alcohol harm than their cisgender (cis) counterparts (Gilbert et al. 2018, Connolly and Gilchrist 2020). Despite within-group differences in alcohol use and the type and severity of associated harm by gender identity (Dermody et al. 2022), TGD people, collectively, appear more likely to report more hazardous patterns of use such as frequent heavy episodic drinking and drinking at home alone (Scheim et al. 2016, Connolly et al. 2024a). Accordingly, TGD people often score higher on screening instruments measuring their risk of alcohol dependence (Connolly et al. 2022) and some data suggest a 3-fold greater prevalence of alcohol use disorders among TGD communities relative to their cis peers (Hughto et al. 2021).

Collectively, TGD people also experience more significant acute harm from alcohol, including alcohol-related blackouts or suicidality (Tupler et al. 2017), and are more likely to experience sexual violence while intoxicated (Connolly et al. 2021). Consistent with this harm, a global sample of TGD survey respondents reported a greater desire to seek help and reduce their alcohol use than cis participants (Connolly et al. 2020). However, interpersonal and systemic discrimination in general population clinical services and in peer support limit their ability to manage or reduce their use (Lyons et al. 2015, Matsuzaka 2018, Connolly et al. 2024b). Specialist alternatives to general population services are seldom available (Ji and Cochran 2022, Chapa Montemayor and Connolly 2023).

Minority stress theories suggest that navigating actual or anticipated transphobic discrimination and associated internalized stigma creates psychosocial conditions predisposing the hazardous relationship with and harm from alcohol that many TGD people report (Hendricks and Testa 2012). While there is a wealth of evidence supporting these theories (Pellicane et al. 2023), viewing the behaviour of any marginalized community solely through a deficit lens risks inadvertently stigmatizing and disempowering.

In the face of unprecedented global anti-trans hostility (Restar et al. 2024, Connolly et al. 2025b), TGD communities and individuals display incredible resilience (Puckett et al. 2019), and many lead fulfilled lives, managing their minoritization without hazardous alcohol use (Paceley et al. 2021). Therefore, just as with cis populations, TGD people who use alcohol do so to experience a range of subjective benefits, the diversity of which is not adequately reflected in the extant literature (Davies et al. 2024).

For example, many alcohol-serving venues have historically held great significance for lesbian, gay, bisexual, trans and other sexual and gender minoritized (LGBTQ+) people (Cerezo et al. 2019, Tensley 2019). They provided a place of relative safety for political and social gathering, authentic self-expression and only quite recently declined in number with the recession of many ‘gaybourhoods’. The community connection found in these spaces is one of many possible benefits that TGD people experience from alcohol use. Developing an understanding of these benefits in their entirety is crucial to developing interventions to help people reduce their harm from alcohol by facilitating the substitution of drinking with activities which offer the same pleasures but confer fewer risks (Morris and Davies 2025, Nicholls and Hunt 2025).

This article reports analyses of responses to two open-ended questions from an online survey, which aimed to understand the subjective benefits of alcohol use for the individual and the role of alcohol within TGD communities in the UK (Davies et al. 2024, Connolly et al. 2024b, 2024a).

## Materials and Methods

### Study design

This study was conducted according to a prespecified protocol (https://osf.io/zgyq7/), and its methods are outlined fully in prior publications (Davies et al. 2024; Connolly et al. 2024b, 2024a). Briefly, an online cross-sectional survey was co-produced with community partners and open for responses from 1 February until 31 March 2022. Recruitment assets directing interested parties to a study website were disseminated on social media by the first and last authors, a community advisory group (CAG), and LGBTQ+ sector organizations. Qualtrics provided an anonymous survey platform. The eligibility criteria were (i) ≥18 years, (ii) trans, non-binary, genderqueer, or gender non-conforming identity, (iii) UK resident, and (iv) current or former alcohol use. A list of TGD-friendly helplines was provided for respondents who found the survey distressing. Respondents could participate in a raffle for vouchers to be used at an LGBTQ+ community bookshop.

Respondents were included in this analytic sample if they responded to either of the following open-ended survey items developed in collaboration with the CAG: (OQ1) ‘Many people drink alcohol as they experience pleasures and benefits from doing so. If you would like to tell the researchers about any benefits you have experienced as a result of drinking alcohol, then please use the box below’; (OQ2) ‘In your experience, does alcohol serve any important roles for the trans and non-binary community?’ Participants were also asked about alcohol-related harm, which is reported elsewhere (Davies et al. 2024, Connolly et al. 2024b).

### Measures

Participants were asked ‘What is your gender identity? Use the free-space option, if required’ and could select one or more of the following responses: ‘Man (including trans man)’, ‘Woman (including trans woman)’, ‘Non-binary’, ‘Genderqueer’, ‘Other gender identity. Please self-describe’. The heterogeneity in responses required respondents to be categorized into one of five analytic groups: ‘man only’, ‘woman only’, ‘non-binary and/or genderqueer’, ‘other gender identity’ and ‘multiple gender identities’. The sample was characterized with questions assessing birth-assigned sex, sexual orientation, ethnicity, formal education level, employment, and whether participants were intersex or neurodivergent. The Alcohol Use Disorders Identification Test (AUDIT) characterized the risk of alcohol-related harm (Babor et al. 2001), adapting the third item in line with advice from the CAG: ‘How often do you have six or more units on one occasion?’. This decision was based on the understanding that sex-specific thresholds may not be valid in this population who may alter one or more sex characteristic to affirm their gender. Kessler-6 (scores ≥13: severe distress) and University of California Los Angeles Loneliness Scale (scores ≥6: significantly lonely) questionnaires measured current psychological distress and loneliness, respectively (Kessler et al. 2002, Hughes et al. 2004).

### Analysis

NVivo 14 was used for data management (Lumivero 2023). Reflexive thematic analysis was chosen to identify and analyse patterns within the data (Braun and Clarke 2019). The six steps of thematic analysis were followed: (i) Authors 1 and 2 became familiar with the data and identified important quotes, (ii) after examining the dataset and identifying recurring patterns, Authors 1 and 2 created codes and assigned segments of the data to them, (iii) Authors 1 and 2 clustered similar codes together before proceeding to generate initial themes, (iv) Authors 1, 2, and 3 reviewed initially generated themes and considered their quality and whether there is enough meaningful data to support them whilst discarding others, (v) Authors 1 and 2 defined and renamed final generated themes, and reconsidered the overall story of the analysis, and (vi) Authors 1 and 2 produced a report consisting of final themes, analytic commentary and data extracts, illustrating each theme with the most compelling examples of data. Quotes were followed with the respondent’s ID, gender identity, age (if known), and alcohol risk category: low risk (AUDIT score: 1–7), increasing risk (8–15), higher risk (16–19), and possible dependence (≥20: Babor et al. 2001).

### Ethics committee review

Oxford Brookes University Research Ethics Committee (191269) reviewed this study.

### Reflexivity/positionality statement

Each author had used alcohol, and several authors have experienced varying degrees of alcohol-related harm. The authors are predominantly white and based in England. The team was diverse in gender identity and modality (four authors are trans or non-binary). This likely afforded the authors greater insight into the findings than a predominantly cis team. Authors 1 (non-binary) and 2 (trans man) reflected on how their experiences could have influenced their interpretation of the data. The team had reflective discussions before agreeing on the interpretation presented here.

## Results

### Characteristics of participants

The study sample comprised 565 participants. Of these, 295 responded to at least one of OQ1 (*n* = 241) and OQ2 (*n* = 214) and were included in this sub-sample. Since individual responses frequently contained data relevant to both research questions, all 455 responses were pooled for analysis. The characteristics of the study sample, published in detail elsewhere (Connolly et al. 2024b), did not differ significantly from this sub-sample (Table 1). Respondents were mostly white, based in England, employed, highly educated and diverse in gender identity and sexual orientation. Respondents reported considerable distress, loneliness, and limited fulfilment of their medical gender-affirmation goals.

**Table 1:** Sample characteristics (*N* = 295)

Variable		*n* (%)
Age [mean (SD), range]	*Missing*	30.0 (10.0), 18–76 *96*
Gender identity	Non-binary and/or genderqueer	116 (39.3)
	Woman only	83 (28.1)
	Multiple gender identities	57 (19.3)
	Man only	33 (11.2)
	Other identity	6 (2.0)
Do you identify as intersex?	No	282 (95.9)
	Prefer not to say	9 (3.1)
	Yes	3 (1.0)
	*Missing*	*1*
Sexual orientation^a^	Bisexual/pansexual	186 (63.1)
	Queer	132 (44.7)
	Gay/lesbian/homosexual	88 (29.8)
	Asexual	41 (14.2)
	Demisexual	30 (10.2)
	Heterosexual	14 (4.7)
Ethnic group	Mixed/multiple ethnic groups	14 (4.7)
	Other (self-describe)	11 (3.7)
	Asian/Asian British	2 (0.7)
	Latino	2 (0.7)
	Black/African/Caribbean/Black British	1 (0.3)
	White	265 (89.8)
Education level	Undergraduate	101 (34.2)
	A-level or equivalent	98 (33.2)
	Postgraduate	72 (24.4)
	GCSE or equivalent	20 (6.8)
	No qualifications	2 (0.7)
	Level 1 or below	1 (0.3)
	*Missing*	*1*
Occupation status	(Self-)employed full-time	132 (44.7)
	Student full-time	63 (21.4)
	(Self-)employed part-time	38 (12.9)
	Not currently employed, studying or caring (not COVID-19-related)	35 (11.9)
	Other (e.g. retired, volunteering, student full-time and employed part-time)	16 (5.4%)
	Caring for dependents children, relatives or other people	5 (1.7)
	Student part-time	3 (1.0)
	Not currently employed, studying or caring (COVID-19-related)	3 (1.0)
Country of residence	England	251 (85.1)
	Scotland	31 (10.5)
	Wales	13 (4.4)
Do you consider yourself to be neurodiverse?	Yes	176 (59.7)
	No	100 (33.9)
	Prefer not to say	18 (6.1)
	*Missing*	*1*
UCLA Loneliness Scale	6–9 (significantly lonely)	225 (76.5)
	*Missing*	*1*
Kessler-6	≥13 (severe distress)	157 (53.2)
‘I have achieved my gender-affirmation goals’	Strongly disagree	97 (32.9)
	Somewhat disagree	92 (31.2)
	Somewhat agree	61 (20.7)
	Neither agree nor disagree	28 (9.5)
	Strongly agree	14 (4.7)
	Not applicable	3 (1.0)
Had difficulty accessing gender-affirming intervention	Yes	201 (68.1)
	No	94 (31.9)

GCSE, general certificate of secondary education; UCLA, University of California Los Angeles. ^a^Total exceeds 100% because respondents could provide more than one response.

### Summary of thematic analysis

**Figure 1:**
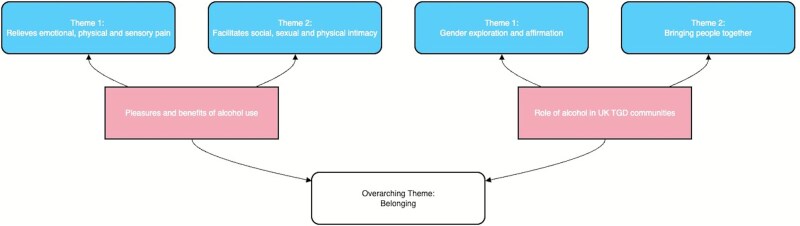
Schematic of thematic synthesis

Figure 1 presents the names and interrelationships of themes developed to capture the most salient patterns in the data. The themes answer the two research questions separately but can also be understood to represent four ways in which alcohol fosters a sense of belonging and community connection for TGD people. For example, through relieving distress, alcohol allowed respondents, who otherwise may be excluded due to gender dysphoria or sensory overload, to ‘function’ and participate in society.

#### Pleasures and subjective benefits of alcohol use for transgender and gender diverse individuals in the UK

Participants experienced a wide range of pleasures and benefits from alcohol. Some of these, such as the taste or ‘buzz’ it creates, are likely shared with the general population. However, participants’ accounts also revealed distinct ways in which alcohol use was embedded within their social, emotional, and embodied experiences as TGD people. Two themes were created to represent data related to alcohol’s pleasures and benefits for the individual: (i) alcohol as a facilitator of social, sexual, and physical intimacy, (ii) alcohol as a means of relieving emotional, physical, and sensory pain. These themes illustrate how alcohol served both to enhance moments of closeness and to ease experiences of discomfort or distress.

##### Alcohol as a facilitator of social, sexual, and physical intimacy

Respondents cited alcohol’s disinhibitory properties as a key benefit of drinking. Alcohol was described as a ‘*social lubricant*’ or ‘*ice breaker*’ (P231, genderqueer, 61 years, low risk) that allowed them to connect deeply with others when they might have otherwise struggled. One participant shared ‘*…lowering of inhibitions can also lead to some of the more profound/open/therapeutic extended discussions I’ve ever had with close friends*’ (P282, multiple gender identities, 32 years, low risk). This was because for some, ‘*alcohol was a useful way of managing…social anxiety*’ (P302, multiple gender identities, low risk) and enhancing self-confidence. Others specifically attributed neurodivergence as the cause of their social challenges and alcohol as a necessary facilitator: ‘*I’m autistic, for a lot of my life alcohol was the only way I was able to cope with socialising / social occasions*’ (P185, non-binary, 25 years, low risk).

Alcohol also permitted TGD people to be more physically close with others. For one respondent, ‘*alcohol makes [them] feel more comfortable/gives [them] an excuse to physically lean on someone, just for human contact, not necessarily romantic or anything*’ (P292, multiple gender identities, 22 years, low risk). Some responses described how alcohol improved their confidence to pursue sexual experiences, allowing them to ‘*feel less awkward*’ or making it ‘*… easier to approach people for sex eg in a bar*’ (P176, non-binary, 23 years, possible dependence). Others shared alcohol-related sexual enhancement that was attributed to lessened bodily discomfort or gender dysphoria, allowing them to connect: ‘*it has always made sex less awkward for me as I’m not very comfortable with my body*’ (P68, woman only, 23 years, increasing risk).

##### Alcohol as a means of relieving emotional, physical, and sensory pain

Respondents often characterized alcohol as medicinal, recounting how its analgesic properties allowed them to function day-to-day. Some reported using alcohol to relieve themselves of various physical pains, including that experienced by transmasculine individuals who bind their chest: *‘if I was going out when I was binding, alcohol helped me forget the physical pain I was in so I could bind all night’* (P281, multiple gender identities, 28 years, low risk)*.* A few respondents, in a majority neurodivergent sample, reported drinking to cope with sensory overload: ‘*I’m also fairly sensitive to loud rooms and lights and get overwhelmed easily but get me a couple of pints in and I can vibe with the best of them*’ (P276, multiple gender identities, 19 years, higher risk).

For most respondents, alcohol was found to soothe otherwise overwhelming emotional pain, fostering an *‘ability to cope rather than doing anything potentially harmful’* (P6, man only, possible dependence). Indeed, there were several accounts of alcohol serving as a harm reduction tool for those experiencing self-injurious or suicidal thoughts: *‘it felt good and stopped me wanting to kill myself temporarily’* (P39, woman only, low risk)*.* Gender dysphoria, in the context of near-inaccessible gender-affirming and mental healthcare, was discussed by several respondents as a precipitant of their distress or suicidality. Table 2 presents a selection of quotes illustrating the role of alcohol in coping with gender dysphoria and limited access to healthcare.

**Table 2:** Respondent quotes illustrating alcohol’s role in managing gender dysphoria and distress associated with inaccessible gender-affirming healthcare

Verbatim quote	Respondent characteristics
All my trans friends (myself included) have been drinking from at least age 14, it helps us forget or at least ignore dysphoria and the thoughts and feelings associated with it	P306, multiple gender identities, age unknown, increasing risk
Being drunk can make me […] worry less about the physical aspects of my body that give me dysphoria. I feel so in the moment that I feel almost removed from my body and my voice	P169, non-binary, 22 years old, increasing risk
In general when I am drunk the disconnect[ion]s between me, my identity, how I am socially viewed, and my physical body shrinks somewhat which is a comforting feeling	P53, woman only, age unknown, increasing risk
It’s a coping mechanism like any other drug. Gender affirming services and mental health help are often difficult to access or prohibitively expensive. Half of the trans/gender non-conforming people I know have…substance abuse issues because it’s more accessible than actual help.	P76, woman only, 25 years old, possible dependence
It can help numb thoughts about gender affirming medicine and surgeries being so hard to obtain	P48, woman only, age unknown, low risk
Early on transition I used to drink a lot as well because trans healthcare is so shitty and the waiting lists are so long I would drink to cope while I waited. Now I’ve had HRT/Surgery I don’t drink as much	P67, woman only, 22 years old, increasing risk

#### Alcohol’s role in transgender and gender diverse communities in the UK

A significant proportion of respondents (*n* = 61, 28.5%) could not identify any role that alcohol served in TGD communities. Some argued that the role of alcohol was similar among both cis and TGD communities, while other reports suggested it is ‘*less important for trans communities than it is for other queer communities*’ (P261, multiple gender identities, 30 years, low risk) since ‘*many trans and non-binary people…actively seek spaces which don’t involve drinking alcohol*’ (P82, woman only, 27 years, low risk). Nonetheless, a greater number of respondents described various ways that alcohol-serving venues (and, by proxy, alcohol) bring the community together and create opportunities for gender exploration and affirmation.

##### Bringing people together

Respondents often described alcohol-serving venues as relatively safe spaces where they felt accepted as their authentic selves. Therefore, these venues became an ‘obvious’ place to escape discrimination and othering: ‘*the most easily accessible queer spaces for a lot of people tend to be bars, pubs, or clubs—queer, lesbian, or gay bars, or even punk or rock bars where LGBTQ+ people are more likely to be accepted. There is a desire to go to and be amongst people…will just accept you without (too much) judgement, and for most of us, the obvious place is a bar’* (P155, non-binary, possible dependence). Many TGD individuals seek to work within LGBTQ+ venues in the hope that they will prove a safer working environment where they can avoid discrimination. As one respondent explains: ‘*A lot of gender variant people tend to work in these venues because they’re the only places that will employ visibly queer people, and so there’s a symbiosis that goes on between alcoholic environments and queer acceptance’* (P221, genderqueer, increasing risk).

For many, pubs were a space where TGD people could build a support network during periods of identity exploration and when navigating social and medical transition. These venues are described as more than a preferred location to socialize or work. They are a safety net in an increasingly hostile society: *‘Through going to the pub… helped form a network of friends and lovers, who became my bedrock of support when it came to exploring my gender identity, coming out and starting my transition. I’m not sure where I would be if it weren’t for the gay scene I discovered in my twenties. I didn’t have a family that supported me. My queer family did’* (P317, multiple gender identities, 39 years, increasing risk). Therefore, alcohol serving venues provide a place of relative safety for TGD communities as well as opportunities to find work and a supportive community of people with shared experiences.

##### Gender exploration and affirmation

Alcohol was frequently reported to create circumstances or conditions that affirmed respondents’ gender. For example, one respondent described: ‘*Alcohol let me experience being trans for the first time via heavy drinking…attending a Rocky Horror Picture Show club night with friends*’ (P102, woman only, 34 years, increasing risk). Several participants reported that alcohol’s anxiolytic properties made them ‘*feel less scared of being visibly trans in public spaces’* (P112, woman only, 49 years, low risk) and imbued them with the confidence to be *‘open about [their] identity*’ and ‘*correct…new people…on pronouns’* (P245, multiple gender identities, 19 years, increasing risk). To elaborate, some respondents consume alcohol to be sufficiently disinhibited to present in ways which affirm their gender and assert their identity confidently when faced with misgendering.

#### Belonging

In the context of structural and interpersonal violence, which increasingly excludes TGD people from society, its institutions and social environments, each theme can be viewed as a mechanism through which alcohol fosters belonging within TGD communities and wider society. Through enhancing intimacy or bringing the community together, alcohol can act directly as a catalyst to enhance belonging. When used as a tool to facilitate gender affirmation or manage various types of pain or distress, alcohol may offer the temporary resilience needed to overcome barriers to participation in public life. For one respondent, alcohol lessened the intensity of gender dysphoria, thereby facilitating social inclusion: ‘*especially it helps reduce my dysphoria in social situations like gatherings and parties’* (P78, woman only, 26 years, low risk)*.* LGBTQ+ venues often provide a space where TGD people can explore and present their gender with relative safety while connecting with a community of people with shared minoritization.

## Discussion

### Key findings

Alcohol use among TGD people in the UK served multiple roles beyond providing pleasure, often functioning as a tool for navigating minoritization and enabling social participation. Respondents described two core subjective benefits: alcohol facilitated intimacy (social, sexual, and physical), and provided temporary relief from emotional, physical, and sensory pain, often related to gender dysphoria, neurodivergence, or structural exclusion. While some respondents noted that alcohol held no specific role in TGD communities, others highlighted the role of alcohol-serving venues as relatively safe spaces for connection, employment, and gender affirmation. Across these accounts, alcohol was consistently framed as a means of fostering belonging, either by easing access to community or enabling self-expression in otherwise ‘othering’ environments.

While much of the literature narrowly frames alcohol use as a coping mechanism for transphobic stigma, qualitative data from two studies conducted in Australia move beyond this framing and support the data presented here (Pienaar et al. 2020, Bailey et al. 2024). Substance use was reported to alleviate gender dysphoria in both studies (Pienaar et al. 2020; Bailey et al. 2024), with one participant noting their drinking was heaviest when managing a prolonged wait for gender-affirming hormones (Bailey et al. 2024). Moreover, participants endorsed substance use as an alternative to self-harm (Pienaar et al. 2020; Bailey et al. 2024) and as a facilitator of experiences which ‘profoundly’ affirmed their gender, either through altered self-perception or exploration enabled by disinhibition (Pienaar et al. 2020).

### Implications

For more than a decade, TGD people in the UK have navigated increasingly regressive policies and legislative changes that compromise their health and right to participate in society (TGEU 2024). Most recently, a landmark ruling from the UK Supreme Court mischaracterized ‘sex’ as binary and immutable (Supreme Court UK 2025), and in doing so, has permitted the exclusion of TGD people from single sex spaces congruent with their gender (Equality and Human Rights Commission 2025). The extent to which TGD people are excluded from public life has increased considerably since these data were collected (discussed elsewhere: Connolly et al. 2025a; Connolly et al. 2025b). Therefore, the implications of these findings, outlined in Table 3, are more relevant now as the benefits this community experiences from alcohol use appear to be related to increasingly unmet needs.

**Table 3:** Implications for policy, practice, and research

Focus	Implications
Policy	-Address structural barriers to timely and equitable access to gender-affirming care to reduce the use of alcohol as a coping strategy -Invest in inclusive alcohol-free LGBTQ+/TGD community spaces to reduce reliance on alcohol to find belonging -Develop robust anti-discrimination policies and targeted employment support for TGD individuals to reduce dependence on alcohol-serving venues for employment
Practice	-Make support for those experiencing alcohol-related harm affirming of TGD identities through correct language (name and pronoun use) and assignment to gender-congruent services and facilities -Improve cultural safety of services by educating providers about the sociocultural context of alcohol use in TGD communities -Employ LGBTQ+/TGD peer workers to support individuals and groups of TGD people seeking support
Research	-In-depth/semi-structured interview studies with a diverse sample of participants are required to gain a more in-depth understanding of the role of alcohol in the lives of TGD individuals and communities -The community-reported benefits of co-use of alcohol with other drugs remains unexplored and may inform strategies to reduce harm

### Strengths and limitations

To the authors’ knowledge, this is the first study to explore the benefits that TGD individuals and communities experience from alcohol use. Key strengths of this work include collaboration with TGD communities from conception to dissemination and the large sample recruited. However, respondents were not representative of the UK TGD population, as most reported white ethnicity. Future studies will ensure that adequate resources are dedicated to correcting this bias.

It is noteworthy that 20% (*n* = 59) of this analytic sample were abstinent from alcohol at the time of the survey. However, upon review of the analytic process, their responses carried little weight in coding and synthesis, and their quotes are not used to illustrate themes. In addition, the high prevalence of neurodivergence in the sample confounded efforts to distinguish whether alcohol use for social facilitation was attributable to neurodivergence or adversity related to TGD status.

The use of open-ended survey questions to collect qualitative data has several limitations. Responses to survey questions are relatively brief and may lack detail when compared to what may have been obtained with interviews. Moreover, the researcher cannot ask follow-up questions or seek clarifications. Conversely, this approach permitted the analysis of a much larger range of perspectives than would have been possible with interviews.

## Conclusion

Understanding the nuanced roles of alcohol for TGD individuals is crucial for developing more comprehensive and supportive health and social interventions that address this community’s unique challenges. This study provides novel insights, moving beyond a narrow focus on coping with stigma. As well as serving as a significant tool for navigating minoritization, our findings demonstrate that alcohol fosters social participation within UK TGD communities. Specifically, respondents highlighted alcohol’s ability to facilitate intimacy and provide temporary relief from emotional, physical, and sensory pain, often linked to gender dysphoria or neurodivergence.

Alcohol-serving venues were frequently identified as crucial, relatively safe spaces for connection, employment, and gender affirmation, and alcohol use was consistently framed as a means to foster belonging and enable self-expression in otherwise ‘othering’ environments. The implications of these findings are particularly pertinent given the increasingly regressive policy landscape for TGD people in the UK. Policymakers should view the provision of sober social spaces and community-building activities for TGD people as public health interventions, and researchers should investigate how best to deliver these initiatives to a small and geographically dispersed population.

## Data Availability

Data are available upon reasonable request to the corresponding author.
